# Molecular Diagnostic for Prospecting Polyhydroxyalkanoate-Producing Bacteria

**DOI:** 10.3390/bioengineering4020052

**Published:** 2017-05-25

**Authors:** Eduarda Morgana da Silva Montenegro, Gabriela Scholante Delabary, Marcus Adonai Castro da Silva, Fernando Dini Andreote, André Oliveira de Souza Lima

**Affiliations:** 1 Centro de Ciências Tecnológicas da Terra e do Mar, Universidade do Vale do Itajaí, R. Uruguai 458, 88302-202 Itajaí-SC, Brazil; dudamorgana@gmail.com (E.M.S.M.); gabidelabary@hotmail.com (G.S.D.); marcus.silva@univali.br (M.A.C.S.); 2 Department of Soil Science, “Luiz de Queiroz” College of Agriculture, University of São Paulo, Piracicaba-SP 13418-260, Brazil; fdandreo@gmail.com

**Keywords:** bioprospecting, biopolymer, environmental diversity

## Abstract

The use of molecular diagnostic techniques for bioprospecting and microbial diversity study purposes has gained more attention thanks to their functionality, low cost and quick results. In this context, ten degenerate primers were designed for the amplification of polyhydroxyalkanoate synthase (*phaC*) gene, which is involved in the production of polyhydroxyalkanoate (PHA)—a biodegradable, renewable biopolymer. Primers were designed based on multiple alignments of *phaC* gene sequences from 218 species that have their genomes already analyzed and deposited at Biocyc databank. The combination of oligos *phaCF3/phaCR1* allowed the amplification of the expected product (PHA synthases families types I and IV) from reference organisms used as positive control (PHA producer). The method was also tested in a multiplex system with two combinations of initiators, using 16 colonies of marine bacteria (pre-characterized for PHA production) as a DNA template. All amplicon positive organisms (*n* = 9) were also PHA producers, thus no false positives were observed. Amplified DNA was sequenced (*n* = 4), allowing for the confirmation of the *pha*C gene identity as well its diversity among marine bacteria. Primers were also tested for screening purposes using 37 colonies from six different environments. Almost 30% of the organisms presented the target amplicon. Thus, the proposed primers are an efficient tool for screening bacteria with potential for the production of PHA as well to study PHA genetic diversity.

## 1. Introduction

Bioplastics polymers have emerged as an alternative to the excessive use of polymers from petrochemical origin, which represent a problem in terms of waste management and environmental impact [1,2]. Polyhydroxyalkanoate (PHA) is among these polymers and has attracted increasing attention due to its properties and suitability for biodegradation, as well as its biocompatibility and thermoplastic characteristics [3,4]. These biopolymers accumulate in the cytoplasm of cells in the form of granules due to nutritional limitations that restrict growth [5]. They are generally associated with carbon reserves or excess in the medium, as well as reduced energy equivalents [6]. Although the procedure for the formation and accumulation of biopolymers is well-known, the main impediment to employing biopolymers is the large scale and the high cost of PHA production, which is nine times more expensive than the production of synthetic plastics [7,8]. In this sense, the bioprospection of bacteria capable of producing these biopolymers in greater quantities from the conversion of cheaper and renewable substrates is necessary, aiming a greater production and consequent reduction in cost [9,10,11].

The genes responsible for PHA synthesis can be classified into four different classes, according to the organization of gene locus and the structural and functional properties of enzymes PHA synthase [12,13]. Class I is represented by gene *phaC* of *Cupriavidus necator*, and class II by *Pseudomonas*, where PHA synthase is encoded by *pha*C1 and *pha*C2 [14]. Class III synthase is composed of the genes *phaC* and *phaE* and can be found in the model organism *Allochromatium vinosum* [15]. Class IV synthase is represented by *Bacillus megaterium*, in which the main genes are *phaC* and *phaR* [16]. Among the genes involved in PHA production, *pha*C is the most important since it encodes the key enzyme for PHA synthesis, thus justifying its choice as an indicative of possible producers of these biopolymers [11].

PHA-producing organisms can be identified and evaluated by different methods [17]. Among the traditional methods, the most frequently used are based on microscopy and specific dyes, such as the lipophilic dye Sudan Black B [18], the fluorescent dye from the Nile [19], and the Nile Red dye [20]. The traditional identification techniques require specific conditions for each bacterium and therefore become more laborious, and moreover offer no specificity and may indicate false positives [13,21,22]. In this context, molecular methods appear as an effective tool for the selection and diagnosis of PHA-producing bacteria, for agility of results, ease of handling, and low cost. Among these techniques, polymerase chain reaction (PCR) is simple and efficient for such a diagnosis [23], as it involves the use of specific primers for the locus of the gene responsible for PHA synthase, the biosynthesis of interest [13,21,22,24,25,26,27]. Thus, the present work aims to design primers capable of identifying bacteria that produce different classes of PHAs, as well as the prospection of environments for the pre-selection of the producing organisms and thus the analysis of the environmental diversity of such organisms.

## 2. Experimental Procedures

### 2.1. Bacteria Strains and Media

The reference bacteria strains *Bacillus pumilus* ATCC 14884, *Bacillus thurigiensis var. israelensis* 4Q2-72, *Bacillus megaterium* ATCC 14581, *Bacillus cereus* ATCC 14579, *Chromobacterium violaceum* (CV11), and *Cupriavidus necator* DSM 545, were used as positive controls for the presence of the gene *pha*C and a negative control was made by using DNA from *Escherichia coli* DH5α. Genomic DNA was isolated (DNeasy Blood & Tissue Kit, Qiagen, Hilden, Germany), quantified after agarose gel electrophoresis (Kodak 1D v.3.5.5b, Kodak, Rochester, NY, USA), and used as a DNA template for PCR (approximately 50 ng per reaction).

Genomic DNA from twelve marine bacteria, *Pseudomonas* sp. (LAMA 572), *Halomonas hydrothermalis* (LAMA 685), *Micrococcus luteus* (LAMA 702), *Brevibacterium* sp. (LAMA 758), *Halomonas* sp. (LAMA 761), LAMA 677, LAMA 726, LAMA 729, LAMA 737, LAMA 748, LAMA 760, LAMA 765, LAMA 790, and LAMA 896, previously recognized (tested by staining with Nile Red in seven culture media) as PHA producers, were used in the developed PHA PCR. Also, negative marine bacteria were applied; *Idiomarina loihiensis* and *Terribacillus saccharophilus*. To check the efficiency in pre-selection of PHA-producer bacteria isolated from the environment, 37 newly isolated bacteria from soils were tested. Genomic DNA of these bacteria was obtained as described, and similarly used for amplifications.

### 2.2. Design and Evaluation of Primers for the Amplification of the Gene phaC

The protocol for primer design was similar to that previously described Lima & Garcês [28]. A total of 218 sequences of the superfamily *pha*C gene were retrieved from the BioCyc Database Collection [29] and analyzed in Megan 4 program [30]. Sequences were aligned and phylogeny was inferred on the basis of neighbor-joining trees built from a similarity matrix determined by the Kimura-2 parameter. The sequences were also analyzed in the amino acid level, which was used to allocate them into the classes of PHA synthase. This was performed using the tool Conserved Domain search, available on NCBI [31]. The description of conserved regions was also evidenced by sequence alignment using the ClustalW algorithm [32] at Unipro UGENE 1.26.1 [33]. The best regions were selected for the primer design, using as parameters a high degree of identity, regions without gaps, and few degenerate bases.

The primer sequences were determined with the online program OligoAnalyzer version 3.1 (Integrate DNA Technologies, Coralville, IA, USA), in which important parameters for the efficiency of PCR reaction were defined, such as the melting temperature (Tm) and the percentage of C+G. The primers drawn were also evaluated at CLC Genomics Workbench 4.8 (CLC bio, Cambridge, MA, USA), enabling the visualization of which primer would produce more annealing results to all the gene sequences used during primer design. The parameter considered in this evaluation was the possibility of up to two degenerate bases for each primer.

### 2.3. Amplification of *phaC* Gene by PCR

The program used for the amplification of *phaC* gene fragments with all primer combinations was a cycle of 94 °C for 4 min, followed by 35 cycles of 94 °C for 45 s, 61 °C for 20 s, 72 °C for 10 s and a final extension of 72 °C for 2 min. As a template, extracted DNA or bacteria cells isolated from the environment were used for *phaC* amplification. Once the best set of primers was established, the program used for *phaC* gene amplification using reference strains was adjusted to one cycle of 94 °C for 4 min, followed by 10 cycles of 94 °C for 30 s, 68 °C for 20 s, as well as 25 cycles of 94 °C for 12 s, 65 °C for 10 s, 72 °C for 7 s and a final extension of 72 °C for 2 min. All reactions were held in the thermocycler Eppendorf Mastercycler Gradient, consisting of 20 μL containing 1× PCR amplification buffer (Invitrogen), 0.2 mM of each dNTP, 0.5 μM of each primer, 1U Taq DNA polymerase (Invitrogen), 2 mM MgCl_2_, and the template DNA. PCR amplicons were observed by electrophoresis in 2% agarose gel further stained with ethidium bromide, and viewed under a UV transilluminator.

### 2.4. DNA Sequencing

The amplified fragments obtained from marine bacteria LAMA 677, LAMA 737, LAMA 748, LAMA 760, and the reference bacteria *C. violaceum* were purified (QIAquick PCR Purification Kit, Qiagen, Hilden, Germany) and sequenced in an ABI-Prism 3100 Genetic Analyzer at ACTGene (Alvorada, RS, Brazil). The identity of the sequences was evaluated through the Genomics Workbench 4.8 program accessing the tool of comparison BLASTX (Nacional Center for Biotechnology Information, Bethesda, MA, USA ) [34]. The gene sequences retrieved by BLAST, in addition to the newly sequenced DNA, were pooled and analyzed for phylogenetic tree classification using multiple alignment calculated by the ClustalW algorithm [33] in Geneious v. 5.5.3 (Biomatters, Auckland, New Zealand).

## 3. Results

### 3.1. Design of Primers for Gene *phaC* Amplification

**Figure 1:**
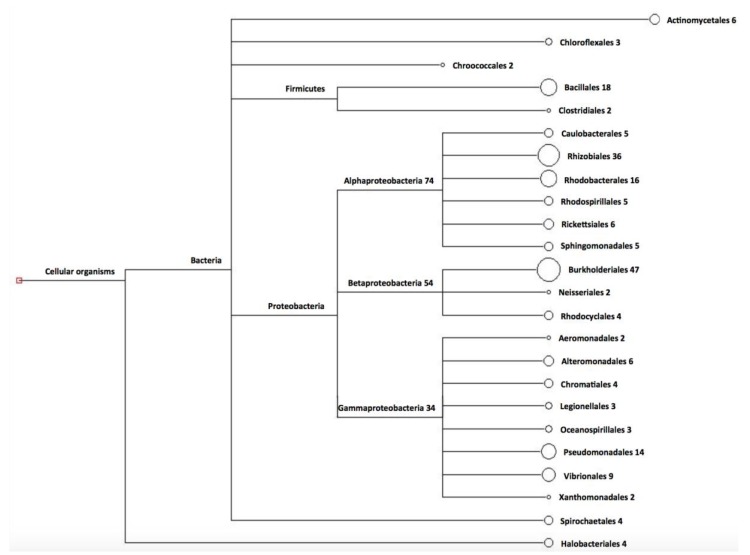
Analysis of the gene sequences used to design the primers, and their phylogenetic classification by the Megan 4 program.

The phylogenetic classification of the 218 sequences of the gene *phaC* (1 sequence = 1 specie) used for primer design indicated a high percentage of organisms belonging to the phylum Proteobacteria (alpha, beta, and gamma) (Figure 1). The phyla Firmicutes and Spirochaetales were also presented, as well as organisms of the orders Chroococcales, Chloroflexales, and Actinomycelates. The domain Archea appears uniquely represented by organisms belonging to the order Halobacteriales (Figure 1). The analysis at the amino acid level showed that the regions of conserved domains were characteristic for three classes of PHA synthases; the classes I, II and III. 

**Figure 2:**
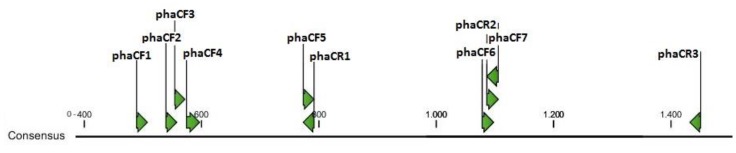
Positioning of the forward and reverse primers accordingly to the consensus sequence.

The application of the designed workflow resulted in the generation of 10 primers, as well as their determined characteristics (sequences, annealing temperatures, relative location to the consensus sequence) and compatibility for annealing with the 218 used sequences (Table 1). Among these, some sets were first selected to amplify the target gene *phaC*. For instance, primers *phaCF3* and *phaCR1* were selected due to their capacity to anneal with a large number of sequences. Also, primer *phaCF1* was picked due to its relative position to *phaCR*1 and its ability to be used in multiplex PCR (Figure 2). These two combinations also resulted in the generation of small fragments; 304 bp for primers *phaCF1/phaCR1* and 239 bp for *phaCF3/phaCR*, which is desired when one is looking for fast detection and maximum amplification efficiency. For a shorter PCR period, amplicons are less likely to vary in size among distinct template sequences. Even so, it is important to consider that amplicon size may vary among different organisms, due to modifications that occurr during evolution. However, variation can be observed, for example, for amplifications with primers *phaCF1/phaCR1*, which resulted in fragments varying from 242 to 316 bp.

### 3.2. Partial Amplification of phaC Gene

**Figure 3:**
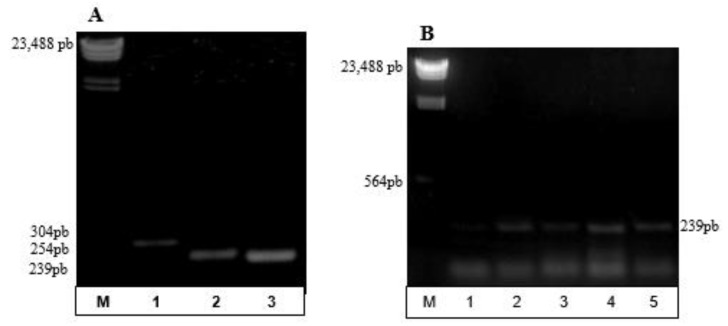

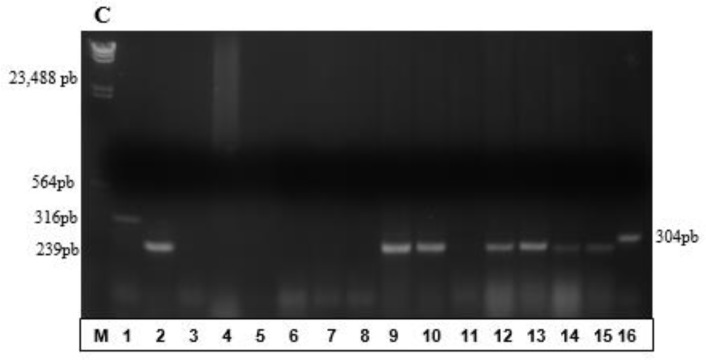
*pha*C gene amplification. Line M: ladder ʎ-hind III. (**A**) *pha*C amplicons for combinations of primers with positive results (organism LAMA 677): 1. *phaCF*1/*phaCR*1; 2. *phaCF*2/*phaCR*1; 3. *phaCF*3/*phaCR*1. (**B**) *pha*C amplicons in: 1. *Bacillus pumilus*; 2. *B. thurigiensis*; 3. *B. megaterium*; 4. *B. cereus* ATCC; 5. *C. necator*. (**C**) *pha*C amplicons in: 1. *Pseudomonas* sp. (LAMA 572); 2. LAMA 677; 3. *Halomonas hydrothermalis* (LAMA 685); 4. LAMA 691; 5. LAMA 694; 6. *Micrococcus luteus* (LAMA 702); 7. LAMA 726; 8. LAMA 729; 9. LAMA 737; 10. LAMA 748; 11. *Brevibacterium* sp. (LAMA 758); 12. LAMA 760; 13. LAMA 790; 14. *Halomonas* sp. (LAMA 761); 15. LAMA 765; 16. LAMA 896.

Although a particular set of primers (*phaCF1, phaCF3*, and *phaCR1*) were revealed to be attractive for *phaC* amplification during in silico analysis, their efficiency and specificity has to be determined in vitro. Therefore, a total of seven pairs of primers were tested using LAMA 677 (PHA producer). Positive results were observed for three of these combinations (Figure 3A), with a remarkable match for functioning of sets previously elected by bioinformatics tools. This pair (*phaCF3/phaCR1*) was further tested using genomic DNA from already known *phaC* carrier species: the model organisms *B. pumilus* (ATCC 14884), *B. thuringiensis* var. israelensis (4Q2-72), *B. megaterium* (ATCC 14581), *B. cereus* (ATCC 14579), and *C. necator* (DSM 545). The expected fragments of 239bp were generated for all organisms tested (Figure 3B) and no amplicon was obtained from the negative control *E. coli* DH5α (data not shown).

Once it was verified that primers were efficient in recognizing PHA-producing bacteria, they were also tested for the detection of potential new polymer producers isolated from environmental samples. For this purpose, two sets of primers (*phaCF1/phaCR1* and *phaCF3/phaCR1*) were used. The use of these primers in multiplex reactions allows for the increase of coverage for the detection of PHA producers. In this context, 16 marine organisms (14 positive and two negative PHA producers) and 37 environmental isolates (unknown PHA production) were screened. The proposed PCR protocol was able to detect *phaC* in nine marine isolates. No false positives were identified, highlighting the specificity of the primers designed. The positive control *C. violaceum* was amplified efficiently. When applying the *phaC* multiplex-PCR with the 37 environmental isolates, *phaC* amplicons were observed in approximately 30% of them (data not shown), revealing the great potential of this method for the screening of PHA producers. 

### 3.3. DNA Sequencing and *phaC* Gene Identification 

**Figure 4:**
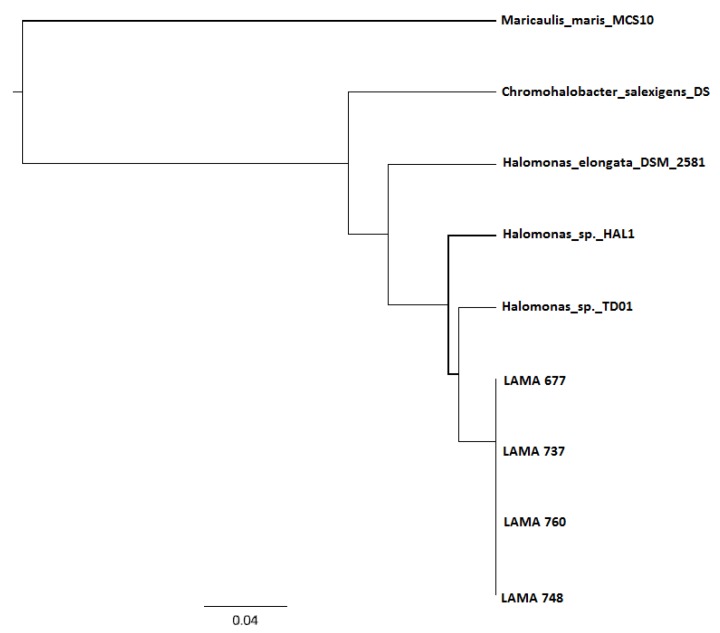
Sub-tree alignment of sequenced amplicons (LAMA 677, LAMA 737, LAMA 748, and LAMA 760), showing organisms with more genetic similarity. The analysis were through the software Geneious V.5.5.3.

Amplicons from different reactions were sequenced and compared to the Genbank database. The sequences identities were compatible with *phaC* genes/proteins previously described. This indicates the specificity and efficiency of the proposed method. The originated sequences also allowed the taxonomic classification of organisms harboring the *phaC* gene (Figure 4). The differential allocation of positive isolates supports the inference that the developed tool is able to detect most of the *phaC* gene diversity that resides in bacterial cells belonging to distinct taxa.

## 4. Discussion 

The use of molecular tools for the detection of organisms with particular features can aid in the field of biotechnology. These methodologies have been used for the determination of the microbial potential to produce PHA at the genetic level as well as to determine how much PHA can be produced by a given organism [17]. Here, we use the same approach to describe newly designed primers for the assessment of bacteria able to produce PHAs. Our approach is based on the growth of records for *phaC* gene sequences, which subsidizes our primer design [17,35]. An innovative aspect is the use of PCR to detect all distinct classes of PHA synthases; Shamala et al. [21] drew primers based only one gene sequence of *B. megaterium*, not obtaining a large breadth of results, and this approach was also employed by Solaiman & Ashby [13], as a result of the simplicity of the method used for primer design. Sheu et al. [24] used the *pha*C gene sequences from 13 gram-positive bacteria to design primers, and presented a greater breadth of results, which were capable of detecting organisms belonging PHA synthase class I and II. The present study used a wide variety of gene sequences belonging to different organisms, resulting in the generation of a precise tool for the detection of organisms with the potential for the production of PHAs from classes I and IV. This method was tested in reference organisms, and was also employed for the screening of new isolates, working in both systems for the detection of the targeted gene.

Colony PCR proved efficient, as this method used amplified regions of interest without necessary DNA extraction methods, which has also been suggested by Sheu et al. [24], making faster work of the screening of environmental organisms. Lane & Benton [23] obtained good results using the same method to determine if six cyanobacteria contained the *phaC* gene. Sasidharam et al. [22] identified the potential of *Vibrio azureus* BTKB33 isolated from marine sediments through PCR confirmation of PHA synthase class I. The use of the PCR technique considerably reduced the number of isolates and thus optimized the process. In addition to the traditional PCR, a multiplex PCR was performed. This methodology used more than one combination of primers to obtain a wider range of results and did not generate false positives, indicating that the use of specific primers for the samples and the chosen conditions were appropriate for the technique [17]. Castroverde et al. [36] showed the efficiency of identifying pathogens using three primers combined in a single PCR. The combination of primers used in the pre-selection of soil organisms in different environments was efficient, and the fragments showed the expected size in approximately 30% of the isolates. These results show the efficiency of using primers designed in the pre-selection of bacteria with the potential for PHA production in samples isolated from the environment. Tzu & Semblante [35] proved the efficiency of multiplex PCR by demonstrating that the primer set was more efficient than the primers tested individually, increasing the detection sensitivity of PHA synthases of classes I and II up to almost 90%. Class I and II PHA synthases were detected from alphaproteobacteria, betaproteobacteria, and gammaproteobacteria, indicating the wide diversity of PHA-accumulating bacteria in wastewater treatment from activated sludge.

Molecular detection of genes involved in PHA synthesis also allows for the prospection of PHA-producing organisms, as well as furthers the understanding and study of gene diversity and evolution [37,38]. Discrepancies in the phylogenetic trees for *phaA*, *phaB*, and *phaC* genes of the PHA biosynthesis have led to the suggestion that horizontal gene transfer may be a major contributor for their evolution [39]. In this way, the use of degenerate primers to study the genetic diversity of genes of biotechnological interest has been gaining prominence, as it aims to define the knowledge of conserved and variable regions of the gene, as well as the structural and functional organization of the enzyme. In the work described by Cheng et al. [40], degenerate primers are used to study the diversity of the subtilase gene with metagenomic DNA samples. This also indicates the potential use of primers in the study of environmental samples taken directly, through DNA metagenomics, which allows to access much of the genetic diversity present in the sample, since organisms that would not be cultivated in the laboratory can be studied directly by the DNA present in the sample. Tai et al. [41] successfully used a culture-independent approach for the detection of the presence of *phaC* genes in limestone soil using primers targeting the class I and II PHA synthases, reassuring the relevance of the approach used in our study.

The related sequences found in studies of diversity still have the potential to be used in genetic improvement programs by site-directed mutations, such as the DNA shuffling technique. Wang et al. [42] pointed out the efficiency of the variant technique called DNA family shuffling for metagenomic studies of homologous genes with specific primers, showing yet another possible application for the primers designed in the present study.

## 5. Conclusions

This study presents a powerful molecular tool for the identification and bioprospecting of bacteria that have the potential to produce PHAs. The tool also shows high potential for the identification of marine bacteria and pre-screening of environmental bacteria that have *pha*C gene, as well as for use in analyses of environmental diversity. 

## Figures and Tables

**Table 1:** Primers designed showing: nomenclature, nucleotide sequences, melting temperatures, initial and final positions correspondent to the consensus sequence and the number of aligned sequences according to the database of organisms used in the study.

Primer ID	Sequence	Tm	Consensus Position	Aligned Sequences *
*phaCF*1	5′TGATSSAGCTGATCCAGTAC3′	53.9°	489–508	18
*phaCF*2	5′CCGCTGCTGATCGTBCCGCC3′	65.5°	539–558	41
*phaCF*3	5′CCGCCSTGGATCAACAAGT3′	58.0°	554–572	61
*phaCF*4	5′CTACATCCTCGACCTGMAGCCGGA3′	63.1°	574–597	24
*phaCF*5	5′GGCTACTGCRTCGGCGGCAC3′	65.1°	773–792	47
*phaCF*6	5′TGGAACDSCGACDCCACCAAC3′	61.6°	1078–1098	0
*phaCF*7	5′CGACRCCACCAACMTGCCGGG3′	65.8°	1086–1106	4
*phaCR*1	5′GTGCCGCCGAYGCAGTAGCC3′	65.1°	773–792	47
*phaCR*2	5′CCCGGCAKGTTGGTGGYGTCG3′	65.8°	1086–1106	4
*phaCR*3	5′CAGTSCGGCCACCAGSWGCC3′	66.3°	1432–1451	0

* Considering a maximum of two mismatches.

## References

[1] Song J.H., Murphy R.J., Narayan R., Davies G.B.H. (2009). Biodegradable and compostable alternatives to conventional plastics. Philos. Trans. R. Soc. Lond. B Biol. Sci..

[2] Barnes D.K.A., Galgani F., Thompson R.C., Barlaz M. (2009). Accumulation and fragmentation of plastic debris in global environments. Philos. Trans. R. Soc. Lond. B Biol. Sci..

[3] Steinbüchel A., Füchtenbush B. (1998). Bacterial and other biological systems for polyester production. Trends Biotechnol..

[4] Rehm B.H. (2007). Biogenesis of microbial polyhydroxyalkanoate granules: A platform technology for the production of tailor-made bioparticles. Curr. Issues Mol. Biol..

[5] Bugnicourt E., Cinelli P., Lazzeri A., Alvarez V.A. (2014). Polyhydroxyalkanoate (PHA): Review of synthesis, characteristics, processing and potential applications in packaging. Express Polym. Lett..

[6] Godbole S. (2016). Methods for identification, quantification and characterization of polyhydroxyalkanoates. Int. J. Bioassays.

[7] Khardenavis A.A., Kumar M.S., Mudliar S.N., Chakrabarti T. (2007). Biotechnological conversion of agro industrial wastewaters into biodegradable plastic, poly-β-hydroxybutyrate. Biosour. Technol..

[8] Tan G.Y.A., Chen C.L., Li L., Ge L., Wang L., Razaad I.M.N., Li Y., Zhao L., Mo Y., Wang J.-Y. (2014). Start are search on biopolymer polyhydroxyalkanoate (PHA): A review. Polymers.

[9] Lee S.Y., Choi J., Wong H.H. (1999). Recent advances in polyhydroxyalkanoate production by bacterial fermentation: Mini-review. Int. J. Biol. Macromol..

[10] Khanna S., Srivastava A.K. (2005). Recent advances in microbial polyhydroxyalkanoates. Process Biochem..

[11] Silva A.L., dos Santosa E.C., dos Santosa Í.A., Lópeza A.M. (2016). Seleção polifásica de microrganismos produtores de polihidroxialcanoatos. Quim. Nova..

[12] Rehm B.H. (2003). Polyester synthases: Natural catalysts for plastics. Biochem. J..

[13] Solaiman D.K., Ashby R.D. (2005). Rapid genetic characterization of poly(hydroxyalkanoate) synthase and its applications. Biomacromolecules.

[14] Hein S., Paletta J.R., Steinbüchel A. (2002). Cloning, characterization and comparison of the *Pseudomonas mendocina* polyhydroxyalkanoate synthases *Pha*C1 and *Pha*C2. Appl. Microbiol. Biotechnol..

[15] Yuan W., Jia Y., Tian J., Snell K.D., Muh U., Sinskey A.J., Lambalot R.H., Walsh C.T., Stubbe J. (2001). Class I and III polyhydroxyalkanoate synthases from *Ralstonia eutropha* and *Allochromatium vinosum*: Characterization and substrate specificity studies. Arch. Biochem. Biophys..

[16] McCooL G.J., Cannon M.C. (2001). *Pha*C and *Pha*R are required for polyhydroxyalkanoic acid synthase activity in *Bacillus megaterium*. J. Bacteriol..

[17] Koller M., Rodríguez-Contreras A. (2015). Techniques for tracing PHA-producing organisms and for qualitative and quantitative analysis of intra-and extracellular PHA. Eng. Life Sci..

[18] Murray R.G.E., Doetsch R.N., Robinow C.F. (1994). Determinative and cytological light microscopy. Am. Soc. Microbiol..

[19] Ostle A.G., Holt J.G. (1982). Nile Blue A as a fluorescent stain for polybeta-hydroxybutyrate. Appl. Environ. Microbiol..

[20] Spiekermann P., Rehm B.H., Kalscheuer R., Baumeister D., Steinbüchel A. (1999). A sensitive, viable-colony staining method using Nile red for direct screening of bacteria that accumulate polyhydroxyalkanoic acids and other lipid storage compounds. Arch. Microbiol..

[21] Shamala T.R., Chandrashekar A., Vijayendra S.V., Kshama L. (2003). Identification of polyhydroxyalkanoate (PHA)-producing Bacillus spp. using the polymerase chain reaction (PCR). J. Appl. Microbiol..

[22] Sasidharan R.S., Bhat S.G., Chandrasekaran M. (2016). Amplification and sequence analysis of *pha*C gene of polyhydroxybutyrate producing *Vibrio azureus* BTKB33 isolated from marine sediments. Ann. Microbiol..

[23] Lane C.E., Benton M.G. (2015). Detection of the enzymatically-active polyhydroxyalkanoate synthase subunit gene, *pha*C, in cyanobacteria via colony PCR. Mol. Cell. Probes.

[24] Sheu D.S., Wang Y.T., Lee C.Y. (2000). Rapid detection of polyhydroxyalkanoate accumulating bacteria isolated from the environment by colony PCR. Microbiology.

[25] Solaiman D.K., Ashby R.D., Foglia T.A. (2000). Rapid and specific identification of medium-chain-length polyhydroxyalkanoate synthase gene by polymerase chain reaction. Appl. Microbiol. Biotechnol..

[26] Kung S.S., Chuang Y.C., Chen C.H., Chien C.C. (2007). Isolation of polyhydroxyalkanoates-producing bacteria using a combination of phenotypic and genotypic approach. Lett. Appl. Microbiol..

[27] Desetty R.D., Mahajan V.S., Khan B.M., Rawal S.K. (2008). Isolation and heterologous expression of PHA synthesising genes from Bacillus thuringiensis R1. World J. Microbiol. Biotechnol..

[28] Lima A.O.S., Garcês S.P.S. (2006). Intragenic Primer Design: Bringing Bioinformatics Tools to the Class. Biochem. Mol. Biol. Educ..

[29] Biocyc Database Collection. http://biocyc.org.

[30] Huson D.H., Auch A., Qi J., Schuster S.C. (2011). Megan Analysis of Metagenome Data. Genome Res..

[31] Nacional Center for Biotechnology Information. https://www.ncbi.nlm.nih.gov/Structure/cdd/wrpsb.cgi.

[32] Larkin M.A., Blackshields G., Brown N.P., Chenna R., McGettigan P.A., McWilliam H., Valentin F., Wallace I.M., Wilm A., Lopez R. (2007). ClustalW and ClustalX version 2. Bioinformatics.

[33] Fursov M.Y., Oshchepkov D.Y, Novikova O.S. UGENE: Interactive computational schemes for genome analysis. Proceedings of the Fifth Moscow International Congress on Biotechnology.

[34] Altschul S.F., Madden T.L., Schaffer A.A., Zhang J., Zhang Z., Miller W., Lipman D.J. (1997). Gapped BLAST and PSI-BLAST: A new generation of protein database search programs. Nucleic Acids Res..

[35] Tzu H.Y., Semblante G.U. (2012). Detection of polyhydroxyalkanoate-accumulating bacteria from domestic wastewater treatment plant using highly sensitive PCR primers. J. Microbiol. Biotechnol..

[36] Castroverde C.D.M., San Luis B.B., Monsalud R.G., Hedreyda C.T. (2006). Differential detection of vibrios pathogenic to shrimp by multiplex PCR. J. Gen. Appl. Microbiol..

[37] Sujatha K., Mahalakshmi A., Shenbagarathai R. (2007). Molecular characterization of *Pseudomonas* sp. LDC-5 involved in accumulation of poly 3 hydroxybutyrate and medium-chain-length poly 3-hydroxyalkanoates. Arch. Microbiol..

[38] Aneja K.K., Ashby R.D., Solaiman D.K.Y. (2009). Altered composition of *Ralstonia eutropha* poly (hydroxyalkanoate) through expression of PHA synthase from *Allochromatium vinosum* ATCC 35206. Biotechnol. Lett..

[39] Kalia V.C., Lal S., Cheema S. (2007). Insight in to the phylogeny of polyhydroxyalkanoate biosynthesis: Horizontal gene transfer. Gene.

[40] Cheng X., Gao M., Wang M., Liu H., Sun J., Gao J. (2011). Subtilase genes diversity in the biogas digester microbiota. Curr. Microbiol..

[41] Tai Y.T., Foong C.P., Najimudin N., Sudesh K. (2016). Discovery of a new polyhydroxyalkanoate synthase from limestone soil through metagenomic approach. J. Biosci. Bioeng..

[42] Wang Q., Wu H., Wang A., Du P., Pei X., Li H., Yin X., Huang L., Xiong X. (2010). Prospecting Metagenomic Enzyme Subfamily Genes for DNA Family Shuffling by a Novel PCR-based Approach. J. Biol. Chem..

